# libNeuroML and PyLEMS: using Python to combine procedural and declarative modeling approaches in computational neuroscience

**DOI:** 10.3389/fninf.2014.00038

**Published:** 2014-04-23

**Authors:** Michael Vella, Robert C. Cannon, Sharon Crook, Andrew P. Davison, Gautham Ganapathy, Hugh P. C. Robinson, R. Angus Silver, Padraig Gleeson

**Affiliations:** ^1^Department of Physiology, Development and Neuroscience, University of CambridgeCambridge, UK; ^2^Textensor LimitedEdinburgh, UK; ^3^School of Mathematical and Statistical Sciences and School of Life Sciences, Arizona State UniversityTempe, AZ, USA; ^4^Unité de Neurosciences, Information et Complexité, CNRSGif sur Yvette, France; ^5^Department of Neuroscience, Physiology and Pharmacology, University College LondonLondon, UK

**Keywords:** NeuroML, LEMS, model specification, standardization, API, modeling, Python, SWC

## Abstract

NeuroML is an XML-based model description language, which provides a powerful common data format for defining and exchanging models of neurons and neuronal networks. In the latest version of NeuroML, the structure and behavior of ion channel, synapse, cell, and network model descriptions are based on underlying definitions provided in LEMS, a domain-independent language for expressing hierarchical mathematical models of physical entities. While declarative approaches for describing models have led to greater exchange of model elements among software tools in computational neuroscience, a frequent criticism of XML-based languages is that they are difficult to work with directly. Here we describe two Application Programming Interfaces (APIs) written in Python (http://www.python.org), which simplify the process of developing and modifying models expressed in NeuroML and LEMS. The libNeuroML API provides a Python object model with a direct mapping to all NeuroML concepts defined by the NeuroML Schema, which facilitates reading and writing the XML equivalents. In addition, it offers a memory-efficient, array-based internal representation, which is useful for handling large-scale connectomics data. The libNeuroML API also includes support for performing common operations that are required when working with NeuroML documents. Access to the LEMS data model is provided by the PyLEMS API, which provides a Python implementation of the LEMS language, including the ability to simulate most models expressed in LEMS. Together, libNeuroML and PyLEMS provide a comprehensive solution for interacting with NeuroML models in a Python environment.

## Introduction

In neuroscience, models based on detailed anatomy and electrophysiology have been used for many years to help explore and understand neural systems. Historically, these models have been expressed using a variety of programming languages, tools, and techniques, leading to a high degree of fragmentation (Cannon et al., 2007). In scientific modeling, domain-specific modeling languages have been developed to address this fragmentation, aid with model exchange, and provide language features, such as built-in methods and classes, which simplify modeling in that particular domain. This is achieved by formalizing common concepts with a standardized set of language expressions and rules. Another key benefit of these languages is that they provide a common format that allows different software tools to process the same model description. When considering models of physical systems, scientists tend to think in terms of the relevant core components of those systems (such as neurons, synapses, and ion channels in the case of neural systems) and the interactions among them. Declarative modeling languages are useful for expressing such conceptual models, as they free the modeler from describing the implementation details of the model, allowing them to focus on the scientific problem. NeuroML is a declarative, XML-based model description language for computational neuroscience, which has been developed as part of an international, collaborative initiative (Goddard et al., 2001; Gleeson et al., 2010). In the latest version of NeuroML (version 2.0 or v2), the structure and behavior of ion channel, synapse, cell, and network model descriptions are based on underlying definitions provided in LEMS, a domain-independent language for expressing hierarchical mathematical models of physical entities (Cannon et al., 2012).

One potential limitation of such a declarative model description language is that the commonly used format for serializing such models, XML, can be difficult to read and write and may be overly verbose, especially for large, complex models. There can be difficulties too when a model includes novel mechanisms that were not considered during the design of the description language or when constructing large, repetitive models, which can be expressed more tersely in a procedural language by means of recursions or loops. Here we describe libNeuroML and PyLEMS, which address these issues for the NeuroML and LEMS languages, respectively. A wide range of models, from point neurons to morphologically detailed, conductance based cell and network models, can be created, parsed, and saved using the libNeuroML API. PyLEMS is a Python API for creating and working with models and model components specified directly in LEMS.

While there are a number of Python applications for reading, modifying and writing XML such as lxml (http://lxml.de)—the advantage of libNeuroML and PyLEMS over these more generic tools is that they contain sub-tools and optimizations specific to the modeling of neural systems. Here we present a brief overview of the current state of NeuroML and LEMS, describe the motivation for developing procedural APIs for these languages, outline the design considerations for libNeuroML and PyLEMS, and provide a number of examples of the usage of these libraries. libNeuroML and PyLEMS allow a user to enjoy the benefits provided by the domain-specificity and rigor of NeuroML and LEMS, while facilitating the use of the Python programming language for procedural model descriptions.

## Overview of NeuroML and LEMS

**Figure 1:**
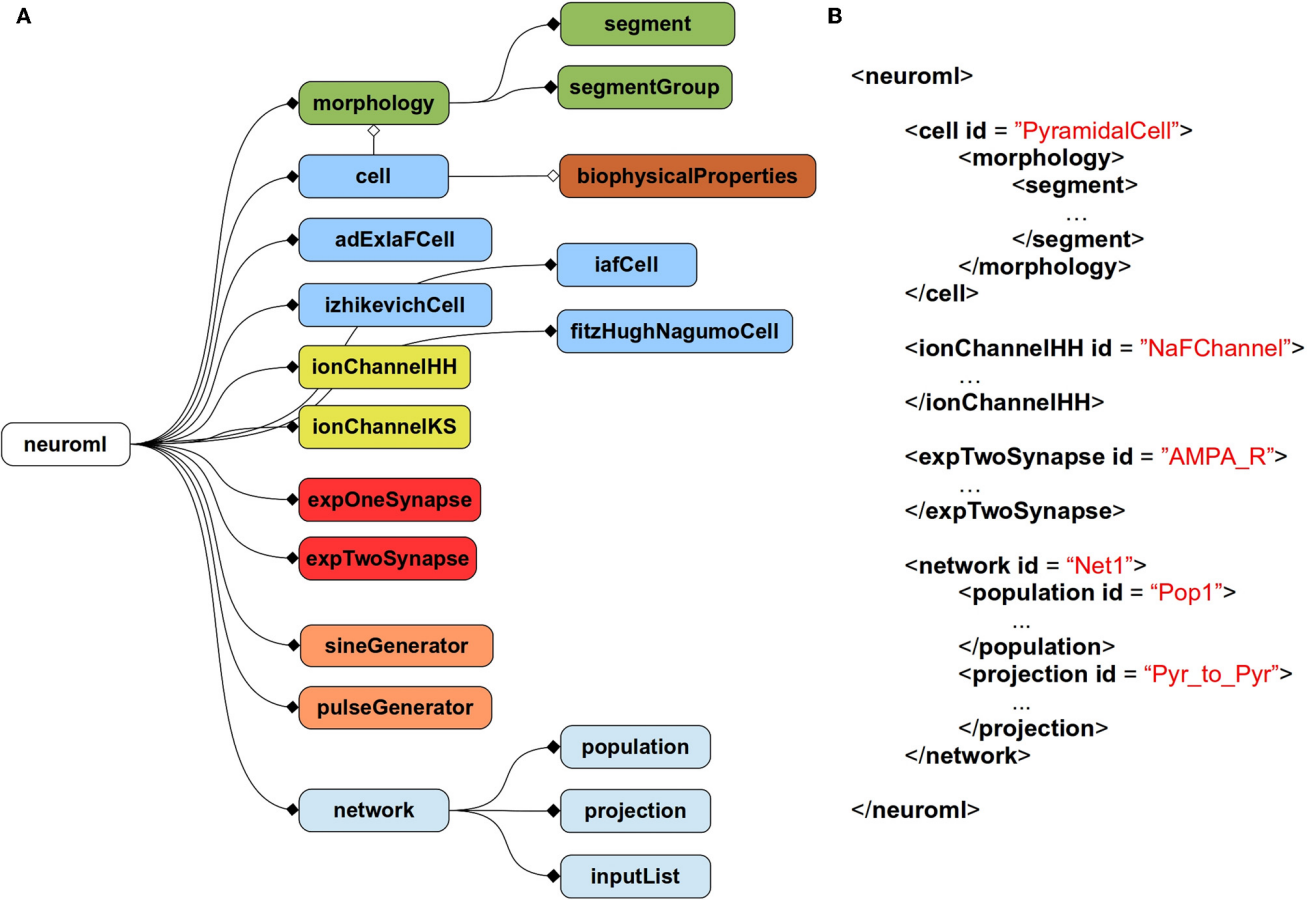
**Structure of NeuroML v2. (A)** Overall structure of NeuroML v2. The top-level element of NeuroML, *neuroml*, contains a number of child elements of various types. A *morphology* element contains lists of *segment* and *segmentGroup* elements defining the structure of the neuronal morphology. Various cell types are allowed including point neurons such as *izhikevichCell*, but also *cell* elements which can have detailed morphologies and *biophysicalProperties* for ion channel densities etc. Ion channels of two main types can be specified, based on either the Hodgkin-Huxley formalism or using a kinetic scheme-based description. Allowed synapse models include single and double exponential conductance waveform models. Current inputs to cells include square pulse and sine waves. Networks contain *populations* of cells, with *projections* between them and lists of inputs. Lines ending in diamonds show containment of elements. Filled diamonds indicate that multiple child elements of that type are permitted, unfilled diamonds indicate only one child of that type is permitted. Not all elements in NeuroML v2 are shown. A full description of all elements in NeuroML v2 is available at http://www.neuroml.org/NeuroML2CoreTypes. **(B)** A partial example of a NeuroML v2 file in XML. This model contains a *cell* with a *morphology*, an ion channel mechanism and a synapse as well as a *network* with a *population* of the cells and a *projection* for synaptic connections between them.

The current scope of NeuroML covers abstract, point neuron models [e.g., leaky integrate and fire models or two-variable spiking neuron models (Izhikevich, 2003; Brette and Gerstner, 2005)], conductance based neuron models, morphologically detailed, multicompartmental neuron models, voltage, and calcium dependent ion channel models, both fixed and plastic synapse models, and models for networks of neurons positioned in 3D with synaptic connections among populations of cells. Figure 1A gives an overview of the elements allowed in a NeuroML file, and Figure 1B shows an example of a NeuroML serialization of a model. NeuroML is being developed by an international consortium of contributors, where the formal specification for the latest version is being developed by the NeuroML Editorial Board (http://www.neuroml.org/editors.php).

**Figure 2:**
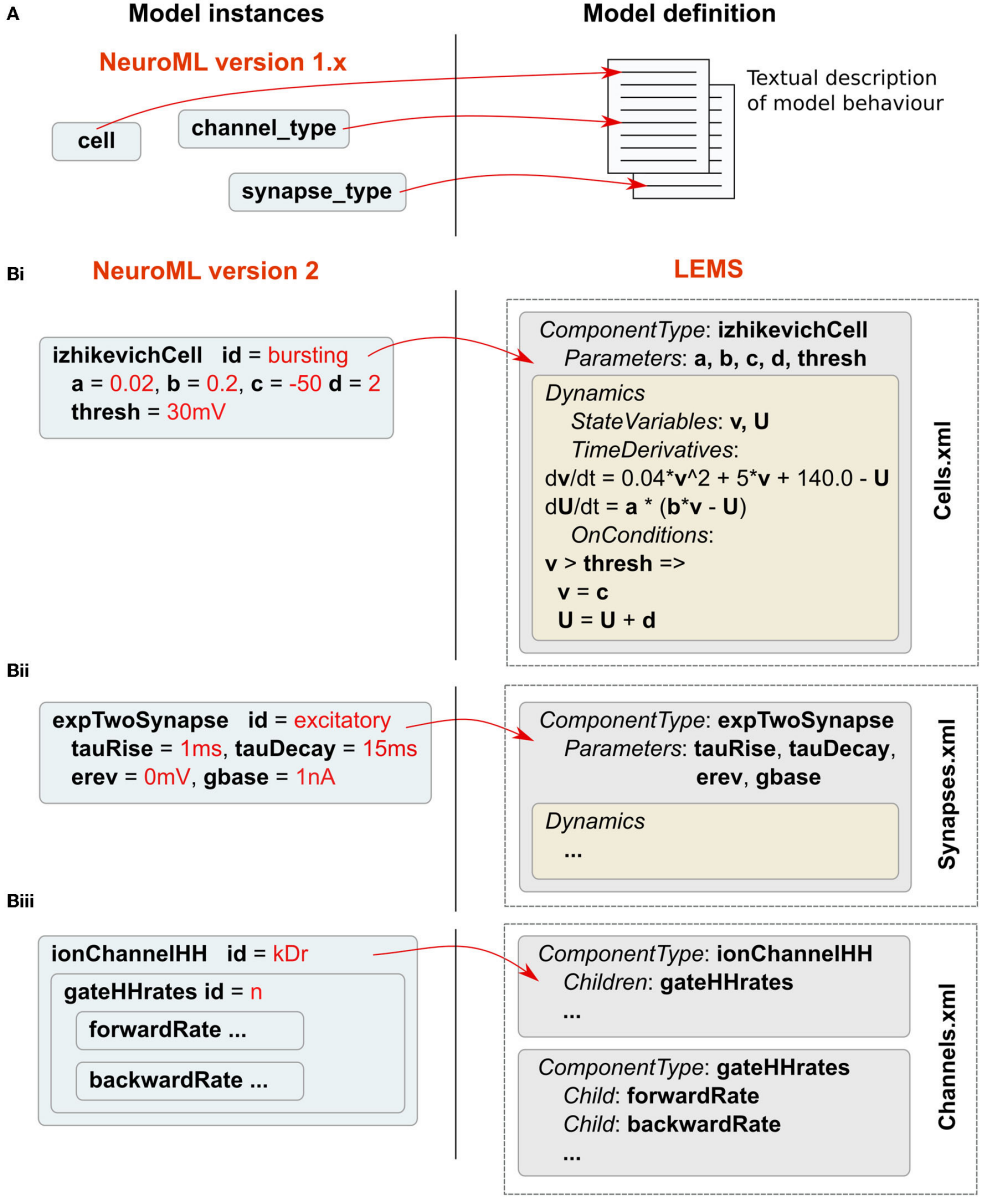
**Relationship between NeuroML v2 and LEMS. (A)** Model definitions in NeuroML v1.x are specified as textual descriptions in human-readable documentation. **(B)** In NeuroML v2, components have a corresponding structural and mathematical definition in LEMS. A number of examples of *ComponentTypes* in LEMS are shown. A *ComponentType izhikevichCell* is defined in LEMS (i), and its parameters are specified as *a, b, c, d*, and *thresh*. The *Dynamics* of the *ComponentType* defines the state variables *v* and *U*. LEMS specifies how these vary with time. Conditions such as when the membrane potential crosses firing threshold are also defined using *OnConditions*. This example of *izhikevichCell* has been simplified to remove scaling factors for unit correctness. Shortened examples of a synapse (ii) and an ion channel model (iii) are also shown. Instances of LEMS *ComponentTypes* can be created by specifying the values for each of the parameters. These instances are represented in NeuroML files. The full NeuroML v2 *ComponentType* definitions are contained in XML files (including Cells.xml, Synapses.xml, Channels.xml as shown here), which have been developed by the NeuroML project and are available at http://www.neuroml.org/NeuroML2CoreTypes.

NeuroML v1.x (Gleeson et al., 2010) focused on conductance-based cell models, often with a corresponding multicompartmental representation of neuronal morphology. For these earlier versions, the mathematical descriptions of model components, such as ion channel models based on the Hodgkin-Huxley formalism (Hodgkin and Huxley, 1952), are specified in user documentation (see supplementary information of Gleeson et al., 2010). Modelers or application developers wishing to utilize or support a feature of NeuroML were required to familiarize themselves with the relevant documentation for that component and ensure compliance for any model description or software application (Figure 2A). A disadvantage of this approach is the possibility for ambiguity in the documentation. NeuroML v2 was designed in conjunction with a new XML-based language called Low Entropy Model Specification language (LEMS), which can be used for creating fully machine-readable definitions of the structure and behavior of the model components (Figure 2B). The elements in NeuroML v2 have corresponding structural and mathematical definitions described in LEMS.

The LEMS language is used to formally describe the components of models of physical systems, which may contain hierarchical relationships. These components can have parameters, which are fixed, and state variables, which vary according to defined relationships. In LEMS, and hence also in NeuroML, all parameters and state variables are dimensional quantities rather than relying on an implicit set of units. Whenever a quantity is expressed in a model, dimensionally correct units must be provided. It is the responsibility of the implementation to check the units and convert to a consistent internal set of units for calculations. Another important concept in LEMS is that of containment of components, encoding the concept that one model element is part of another (e.g., a population of cells is part of a network, a gate is part of an ion channel). Another key LEMS concept is the ability to declare a prototype *ComponentType*, which defines the generic structure and dynamics for a broad class of models. Models can then be instantiated as *Components* by providing a set of parameters for a specific instance. An example of the *ComponentType* concept is shown in Figure 2Bi for the spiking cell model of Izhikevich (2003), a widely-used model in computational neuroscience, which exhibits a diverse range of physiologically-realistic spiking behaviors by changing a small set of parameters in the model. Instances of the model (*Components*), such as the cell defined on the left in Figure 2Bi, are specified by providing specific values for the parameters. NeuroML and LEMS use Fortran-like “gt” and “lt” inequality operator symbols instead of “>” and “<.” This is done because in XML the symbols “>” and “<” are used in the declaration of XML tags.

While any modeler is free to describe a model in LEMS, the NeuroML initiative has developed a set of curated LEMS definitions (e.g., Cells.xml and Synapses.xml in Figure 2B) for commonly used models, which form the basis for NeuroML v2. Similar classes of model types can be linked together by using the type extension mechanism of LEMS. For example, any object which produces a current extends the type *basePointCurrent*, while all synaptic models extend *baseSynapse*. This approach also provides for user defined extensions to the core NeuroML language.

LEMS provides complete, machine readable model definitions for a broad range of cell, ion channel and synapse models in NeuroML v2 (though not yet for multicompartmental cell models, see Discussion). Each NeuroML release includes a W3C XML Schema Document (XSD, http://www.w3.org/XML/Schema), which can be used to validate NeuroML documents, i.e., check whether all required elements and attributes are present. Simulators and other applications that aim to support the language can choose to base their import/export functions on the structure of the language specified by the Schema and associated documentation, as was the case in NeuroML v1.x. However, any simulator utilizing NeuroML should be designed to ensure that simulated behavior complies with LEMS definitions in order for that application to be NeuroML-compliant. The LEMS *ComponentType* definitions are also defined in XML format, and a LEMS-specific Schema document exists for the purpose of validation.

## Declarative and procedural model descriptions in computational neuroscience

Procedural programming languages require a description of the sequence of steps to be executed (or control flow) in a computer program in terms of sequential commands. On the other hand, declarative languages provide the information needed for computation without directly expressing the control flow. In this section the comparative advantages and disadvantages of these paradigms are described in the context of modeling in neuroscience.

### Advantages of declarative model descriptions

Declarative specification of models can be of benefit in three principal respects: model readability, model interoperability/validation, and the avoidance of fragmentation.

Domain-specific declarative languages are generally easier to read and understand because they shift focus onto describing the nature of the problem being solved rather than the details of the specific sequence of operations used to solve that problem. A neuroscientist with a background in biophysics and a minimal amount of programming experience can read, understand and modify a NeuroML model with relative ease. However, this may not be true for an equivalent model written in a general-purpose procedural language such as Python or C. Moreover, declarative formats like XML also allow for an easier transformation into more readable presentations such as HTML websites.

Declarative languages for computational modeling such as NeuroML provide a good interchange format for different software tools by ensuring model completeness and facilitating machine-parsing of the models. The domain-specificity of a particular declarative language means that relationships between model elements can be formalized (e.g., in an XML Schema), providing a fixed framework for defining models. This facilitates model validation and typically makes it simpler to diagnose errors, although it should be noted that this form of validation indicates little about the scientific veracity of the model (Crook et al., 2013).

As a consequence of improved readability and software interoperability, efforts such as NeuroML also reduce the fragmentation in a scientific discipline. The development of common formats for specification of models accelerates progress by encouraging model sharing and reuse, as can be seen with SBML (the Systems Biology Markup Language Hucka et al., 2003 and MathML Miner, 2005).

### Advantages of procedural model descriptions

Despite the advantages of writing models in a declarative manner, procedural specification of models does have two important benefits. First, procedural languages are generally more practical for describing models when there are aspects of the model that can not be described with the base components of a declarative modeling language. When the boundaries and concepts for model design are not clear, a procedural language often will provide a needed degree of flexibility. As an example, if one were designing a multicompartmental model where a particular membrane conductance was only present on dendrites more distal than the second branch point from the soma, it would probably be easier to describe this concisely using a general-purpose language like Python or C, since NeuroML does not natively support defining regions of the cell in this way. A second advantage of developing libraries to allow procedural model description development is that it is easier to integrate with other libraries such as those providing visualization utilities or analysis routines. The libSBML API (Bornstein et al., 2008) for the SBML language has been an important factor in the widespread support for that language among systems biology applications (Sauro and Bergmann, 2008).

Thus, both procedural and declarative paradigms play an important role in computational neuroscience. When working within a widely used modeling formalism, such as Hodgkin-Huxley type conductance based models, having the ability to easily export it to a declarative format is useful and important. In such a situation, declarative modeling allows for ease of development, model interchange among software tools, and model reproducibility. When working with novel modeling approaches or when integrating with scientific or visualization libraries, it is useful to use a procedural approach.

## libNeuroML and PyLEMS

**Figure 3:**
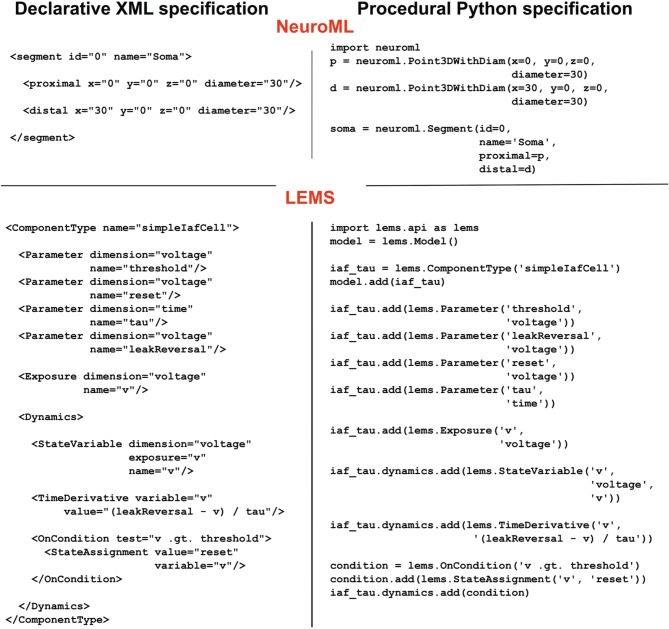
**Examples of NeuroML and LEMS models specified in XML and equivalent models specified with Python using the libNeuroML and PyLEMS APIs, respectively**. The XML code on the left is generated automatically by the Python code on the right. The NeuroML example describes a soma segment as a 3D cylinder. The LEMS example illustrates how a simple integrate and fire cell can be defined.

**Figure 4:**
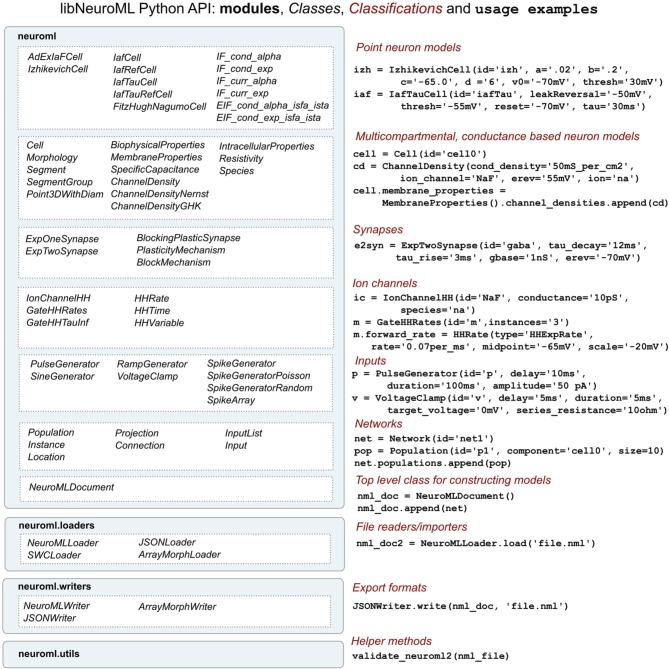
**An overview of the structure of the libNeuroML API**. The API is divided into four main modules (left), the largest of which (neuroml) consists of Python classes generated from the NeuroML XML Schema Document. There is roughly a one to one correspondence between the NeuroML elements and Python classes. These classes can be split into a number of broad classifications (right) based on the types of models they represent. Examples of Python code using the classes is also shown on the right. Extra modules have been created to facilitate loading or writing NeuroML (in XML or other serialization formats) and for validating NeuroML files.

**Figure 5:**
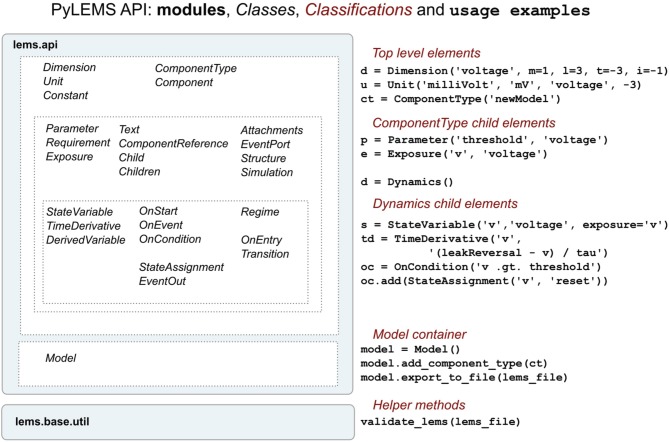
**An overview of the structure of the PyLEMS API**. Classes are present for each of the main elements of the LEMS language (left). Examples of using these in a Python script are shown on the right. The Model class is a container which can be used to hold the model and can export this to an XML file. Generated or other LEMS files can also be validated through the API. More information about the LEMS elements on which the classes above are based can be found here: http://lems.github.io/LEMS/elements.

To facilitate procedural model description development for the NeuroML and LEMS languages we have developed libNeuroML and PyLEMS. These Python modules can be imported into a Python script to allow loading of XML files in their respective formats, parsing and editing of the models using APIs which closely follow the structure of the XML languages, and saving in valid XML. libNeuroML, which parses and saves NeuroML v2, has added functionality to use optimized representations of large models, both internally and as serialization formats. PyLEMS has the additional ability to simulate the dynamical behavior of LEMS models. Figure 3 shows examples of XML from NeuroML and LEMS and the Python code that can be used to create the equivalent entities. More detailed overviews of the APIs for libNeuroML and PyLEMS are shown in Figures 4, 5, respectively. A core aim of libNeuroML and PyLEMS is to provide production-quality, easy-to-use utilities for the manipulation of NeuroML and LEMS, using tools and standards familiar to Python programmers that are also easy to use for those less experienced with Python. With this core aim in mind, libNeuroML and PyLEMS have been implemented with the design goals described below.

### Design goals shared by both libNeuroML and PyLEMS

#### Python naming conventions and adherence to Python PEP8 style guide

libNeuroML and PyLEMS strongly adhere to naming conventions that are widely used in the Python community and codified in the PEP8 Style Guide for Python Code (http://www.python.org/dev/peps/pep-0008/). In the case of libNeuroML, this adherence to convention is enforced by automated conversion from NeuroML to Python naming conventions, which occurs during the automatic generation of the libNeuroML object model from the corresponding NeuroML Schema (see below). NeuroML elements have their names modified in the libNeuroML object model to use standard Python naming conventions; for instance, *izhikevichCell* (element) and *q10Settings* (attribute) in NeuroML become **IzhikevichCell** (class) and **q10_settings** (field), respectively in libNeuroML.

#### Automated XML validation

libNeuroML provides a validation utility to ensure that NeuroML documents are well-formed (following the basic syntactic rules of XML) and valid (following the structure defined in the NeuroML Schema). A similar utility exists in PyLEMS for validating against the LEMS Schema. Note that while the NeuroML *ComponentType* definitions as well as XML generated by PyLEMS are valid according to this Schema, PyLEMS is flexible enough to parse invalid LEMS files (e.g., with reordered elements) as long as they follow the correct containment rules for LEMS elements.

#### Ease of installation

libNeuroML and PyLEMS utilize the standard distutils (http://docs.python.org/2/library/distutils.html) tool for packaging Python programs, making installation standard and simple. Both APIs require the lxml (http://lxml.de) Python package. Additionally, libNeuroML requires numpy (http://www.numpy.org), jsonpickle (https://pypi.python.org/pypi/jsonpickle), mongodb (http://docs.mongodb.org/ecosystem/drivers/python), and PyTables (http://www.pytables.org) packages. Both APIs are currently tested and stable for Python versions 2.6 and 2.7. All of these packages can be obtained from the Python Package Index (https://pypi.python.org/pypi). The full source code for the libraries can be obtained from https://github.com/NeuralEnsemble/libNeuroML and https://github.com/LEMS/pylems.

### Additional design goals for libNeuroML

#### Auto-generation from NeuroML schema

In libNeuroML every element (such as *cell*, *network* or *pulseGenerator*, see Figure 1A) in the NeuroML Schema corresponds to a concrete class and therefore can be instantiated as an object by calling **neuroml.<NameOfClass>()**. This is is possible because the libNeuroML core object model is auto-generated from a NeuroML Schema file via the generateDS tool (https://bitbucket.org/dkuhlman/generateds), where the generated object model defines all necessary type interfaces. Therefore, libNeuroML provides a complete and direct mapping between the NeuroML Schema and its internal Python object model with several advantages:

1. *Maintainability*. libNeuroML can be rapidly updated to reflect the latest NeuroML Schema with little or no knowledge of implementation or architecture. This allows libNeuroML to be a core part of the regular NeuroML release process. New versions of the NeuroML Schema will always be released along with a libNeuroML version which reflects those Schema changes.
2. *Backward—support*. This feature allows users to create a Python API for handling NeuroML even if they are working with older versions of the NeuroML representation format, including versions that existed before libNeuroML. However, the user must auto-generate this libNeuroML version via the generateDS tool as described in libNeuroML documentation.
3. *Flexibility*. It is possible for a user to modify the NeuroML Schema in order to develop new components or change existing ones. A custom copy of libNeuroML can then be generated for further testing. Such modifications can then be proposed for inclusion in the language through discussions on the NeuroML mailing list.
4. *Saving to valid NeuroML*. A key feature of generateDS and, by extension, libNeuroML is the ability to save XML files which are valid against the Schema used to generate the API.
5. *Automatic conversion of names to Python format*. While the convention in NeuroML is to use “camel case” for naming elements and their attributes, the generated Python class and method names automatically are converted to Python naming conventions.

One notable disadvantage of an auto-generated API is that it contains syntax which is less usable than it might be for a “hand-written” API. However, we have contributed updates to the generateDS tool to increase usability and readability of the API, and these will continue to be improved.

#### Serialization and database support

This section describes the different serialization formats provided by libNeuroML. In this section the term NeuroML specifically refers to the XML-based file format rather than NeuroML as a model description language. libNeuroML supports serialization of models into four different storage formats: NeuroML (XML), JavaScript Object Notation (JSON) (http://www.json.org), HDF5 (http://www.hdfgroup.org/HDF5), and SWC (Cannon et al., 1999). Of these four serialization formats, two (NeuroML and JSON) are lossless, which is to say that they preserve all of the NeuroML model data, and the other two (HDF5 and SWC) are lossy and are only able to serialize a subset of the data, namely the morphological structure of detailed neuronal reconstructions. In the case of SWC however, the format is only suited to serializing a small subset of morphological data and is intrinsically unable to serialize complex models, due to the nature of the SWC format as described below.

In addition to file-based serialization, libNeuroML provides the ability to store data in a MongoDB (http://www.mongodb.org) database via an intermediate JSON document. All serialization and deserialization operations, including database operations, are carried out through the **neuroml.writers** and **neuroml.loaders** modules of libNeuroML, respectively (Figure 4).

***NeuroML (XML)***. The de-facto libNeuroML serialization output is the standard XML-based format in which NeuroML models are written. Small to medium-sized NeuroML models generally should be written in this format, as these documents can be edited with standard text editors and validated against the NeuroML Schema independently of libNeuroML. The XML serialization is the format most widely supported by NeuroML compliant software tools (http://www.neuroml.org/tool_support.php). It is recommended that unless there is a clear reason to do otherwise, users should use XML serialization until they encounter performance bottlenecks. NeuroML serialization, though the slowest and least concise, permits the use of NeuroML-compliant tools (such as morphology viewers) and the ability to easily track changes across file versions.

***JSON***. JSON is an open, text-based format for human-readable data interchange. Any file serialized by libNeuroML to JSON format can be loaded by libNeuroML without any loss of information and thus can be re-serialized as NeuroML. The **jsonpickle** (https://github.com/jsonpickle/jsonpickle) module is used to serialize NeuroML documents in JSON format. There are three primary advantages of JSON serialization:

1. Some users report that JSON is easier to read and understand than XML.
2. Many tools exist to facilitate the use of JSON in situations where data is transmitted over networks. This is particularly true for web-based applications since a number of such tools and frameworks are optimized for working with JSON.
3. When used in conjunction with the libNeuroML **arraymorph** module, large-scale morphological reconstructions require substantially less space on disk when stored as JSON-serialized documents.

***SWC***. SWC is a tree-based representation used for storing morphological reconstructions of neurons, and it is the data storage format of the NeuroMorpho.org database of reconstructed neurons (Ascoli et al., 2007). In SWC, each node contains diameter and position information, and the conical frustum between two nodes is treated as a segment of morphology. This allows, for instance, reconstructions of dendritic arborizations of theoretically-unlimited complexity. libNeuroML can import the **Morphology** component of a cell in SWC format. However, any other NeuroML component data such as information about the distribution of channels or synapses with regard to the morphology would be lost when serializing in this format.

***HDF5***. HDF5 (Hierarchical Data Format 5, http://www.hdfgroup.org/HDF5) is a set of file formats and tools for storing and organizing large amounts of numerical and hierarchical data. HDF5 serialization is provided by libNeuroML, although presently the level of serialization support which is provided only extends to morphological reconstructions of neurons. The main advantage of HDF5 support is the relatively low memory footprint and support for rapid read/write operations, which is demonstrated in the benchmarks section of this article.

***MongoDB***. MongoDB (http://www.mongodb.org/) is a document-oriented “NoSQL” database which departs from the traditional table-based relational database paradigm toward JSON-like documents with dynamic schemas. Its document-oriented approach is particularly suitable for storing NeuroML documents. The MongoDB support provided by libNeuroML is particularly useful for users wishing to store large amounts of data on a server, such as for use with a website. Since MongoDB is compatible with NeuroML's **arraymorph** module, large models can be stored in a MongoDB database at a fraction of the disk requirements for equivalent NeuroML documents. One MongoDB limitation is that JSON files exceeding 16 MB can not be stored; large documents (more than 100,000 segments) should therefore be stored as individual files.

***Serialization file sizes***. For reconstructed morphologies, the file sizes of the different serialization formats vary linearly with the number of segments in the morphology with NeuroML requiring 316 B/segment, JSON requiring 62 B/segment, and HDF5 requiring 41 B/segment. For a typical reconstructed morphology of 2000 segments, a NeuroML (XML) file would require 617 KB, and the corresponding JSON and HDF5 files would require 80 and 41 KB, respectively.

### Optimized internal representation (arraymorph)

The **arraymorph** module of libNeuroML provides highly optimized representations of neuronal morphologies to increase read/write speed and reduce memory footprint. Neuronal morphologies are instantiated as a non-NeuroML **ArrayMorphology** type, which inherits from the standard NeuroML **Morphology** type but uses Numpy (http://www.numpy.org/) arrays to represent morphologies in a way that is transparent to the user and is highly-influenced by the SWC format. With the **arraymorph** module, it remains possible to manipulate NeuroML segment objects, and all helper methods and properties continue to work as with the standard NeuroML **Morphology** type. However, low-level access to the arrays is also possible, although the user must understand the internal details of implementation. The rationale for development of this module stems from several drawbacks of both Python and XML that can be summarized as follows:

- Python requires a relatively large memory footprint when instantiating objects. Representing every component in a NeuroML file in-memory by an instantiated object can demand an unfeasibly-large amount of memory. A typical reconstructed morphology of 1600 segments requires 6.9 MB in memory; a network of over 1000 cells would therefore require over 1 GB of memory.
- XML serialization results in performance bottlenecks when read/write operations are conducted on large XML files because NeuroML is a relatively verbose format, and the whole file must be loaded for even one element to be accessed.
- Because many NeuroML documents are mainly morphological reconstructions with a very small amount of metadata, a SWC-like format can store most of the same data with a much smaller performance overhead.
- Recent projects in the field of computational neuroscience such as the Blue Brain Project (Markram, 2006) and the Brain Activity Map Project (Alivisatos et al., 2012) require increasingly large amounts of morphological reconstruction data.
- Some mathematical analysis methods and transformations that can be performed on reconstructed morphologies, such as morphology transpositions or branching analysis, can be performed on SWC-like flat representations more efficiently than by recursive traversals of tree-based data structures.
- An optimized internal morphology offers the advantage of faster read/write operations while simultaneously hiding a lot of the underlying complexity from the user.
- The SWC-like nature of the **ArrayMorphology** class provides a conceptual bridge between the component-based representation idea underlying NeuroML and the node-based morphology representation underlying SWC.

### Additional design goals for PyLEMS

#### Simulation of LEMS models

When defining a new language like LEMS, which aims to encapsulate a broad range of dynamical model descriptions, it is imperative to execute the models and test the results against expected behavior. This has been a core principle for the develoment of jLEMS (https://github.com/LEMS/jLEMS), a Java based reference implementation of LEMS developed in parallel with LEMS. A feature of LEMS is not considered stable by the design team unless an example can be executed with jLEMS. Like jLEMS, PyLEMS is not only an API for the language, but also can be used to simulate the dynamical behavior of the models it parses. While both jLEMS and PyLEMS feature only simple numerical integration techniques (forward Euler and additionally, in the case of jLEMS, Runge Kutta fourth-order), both implementations are required to produce the same model behavior for a given model specification. While some of the advanced features of LEMS (e.g., kinetic scheme based ion channel descriptions) are not yet considered stable in PyLEMS, this dual test for LEMS model behavior is invaluable in the development of the language.

## Methods and results

### Implementation of XML bindings for libNeuroML

The generateDS Python package is used for automatic generation of NeuroML XML-bindings in libNeuroML from the NeuroML Schema. Full details of this conversion process are available in the libNeuroML documentation (http://libneuromldev.readthedocs.org/en/latest/implementation_of_bindings.html).

### Benchmarks

**Figure 6:**
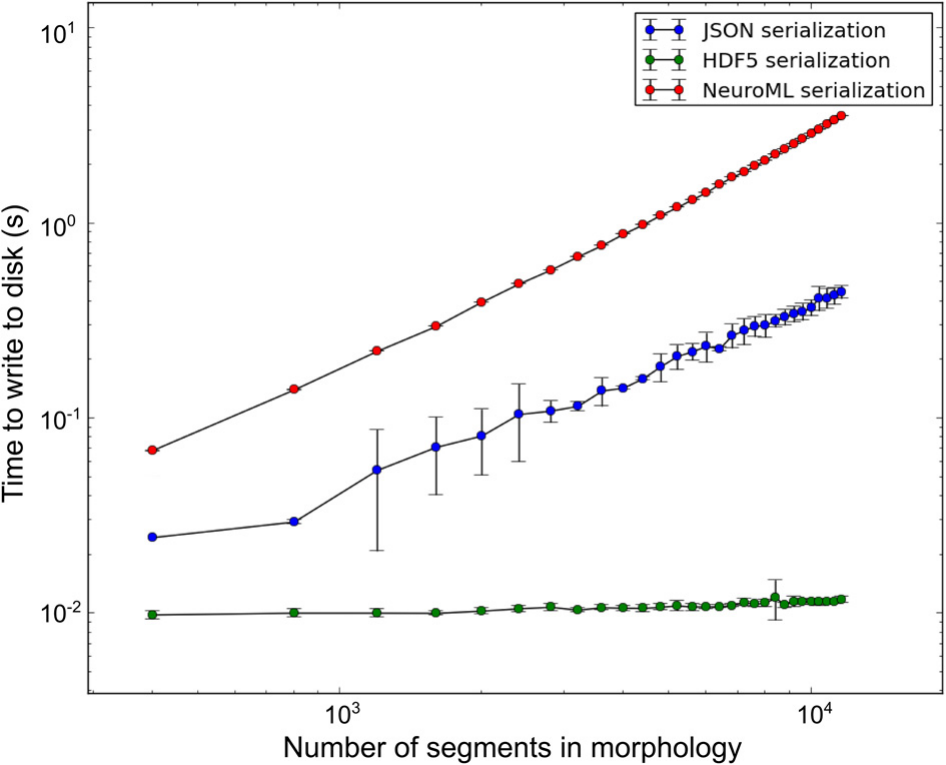
**Disk write time for synthetic morphologies in different serialization formats**. Error bars represent one standard deviation (sample size = 10).

libNeuroML contains a benchmarking module which presently supports benchmarks for writing NeuroML model data to disk in a variety of serializations. All benchmarks described here were run on a DELL PowerEdge R815 containing a 64-core AMD Opteron 6272 processor, 7.2K RPM Hard Drive and 128 GB memory (16 × 8 GB 1600 MHz RDIMMs). During benchmarking a range of synthetic morphologies of various sizes were generated (sample size of 10) using the **arraymorph** module and written to disk. Write-to-disk times were recorded. The benchmark results (Figure 6) indicate that NeuroML (XML) serialization of NeuroML documents with libNeuroML is relatively slow in terms of read/write operations. However, NeuroML documents are readable by humans and a wide variety of software tools, making this serialization format advisable for most use cases. Benchmark results also show that HDF5 write operations are approximately 100 and 600 times faster than corresponding JSON and XML write operations, respectively, suggesting that if performance bottlenecks are encountered, HDF5 should be investigated for storing morphological reconstruction data. JSON serialization offers a good compromise between the speed of writing to disk provided by HDF5 and the readability and full NeuroML language support of XML serialization. The higher write speeds of JSON when compared to XML are achieved due to use of the **arraymorph** module and the fact that JSON-serialized NeuroML is a less verbose than the XML equivalent.

### Testing, validation, and continuous integration

libNeuroML development follows modern software development practices, including a stable release cycle, version control, testing, and continuous integration. Each libNeuroML stable release is part of the official NeuroML release cycle. The git (http://git-scm.com) source code management system is used to provide version control management and is used in conjunction with GitHub (https://github.com) to provide issue tracking functionality as well as a central repository for developers. The package is tested using a variety of unit and integration tests, and the Travis-CI (https://travis-ci.org) continuous integration platform is used to confirm that libNeuroML is correctly installing and that all tests are passed every time a change is applied to the software and pushed to GitHub. Test coverage is 91% [measured with the Python Coverage module version 3.7.1 (https://pypi.python.org/pypi/coverage)]. PyLEMS is also developed on GitHub and released as part of NeuroML release cycle. Basic unit testing and continuous integration on Travis-CI have been added to PyLEMS and will be expanded in the future.

## Usage examples

### Examples using libNeuroML

#### Network example

**Figure 7:**
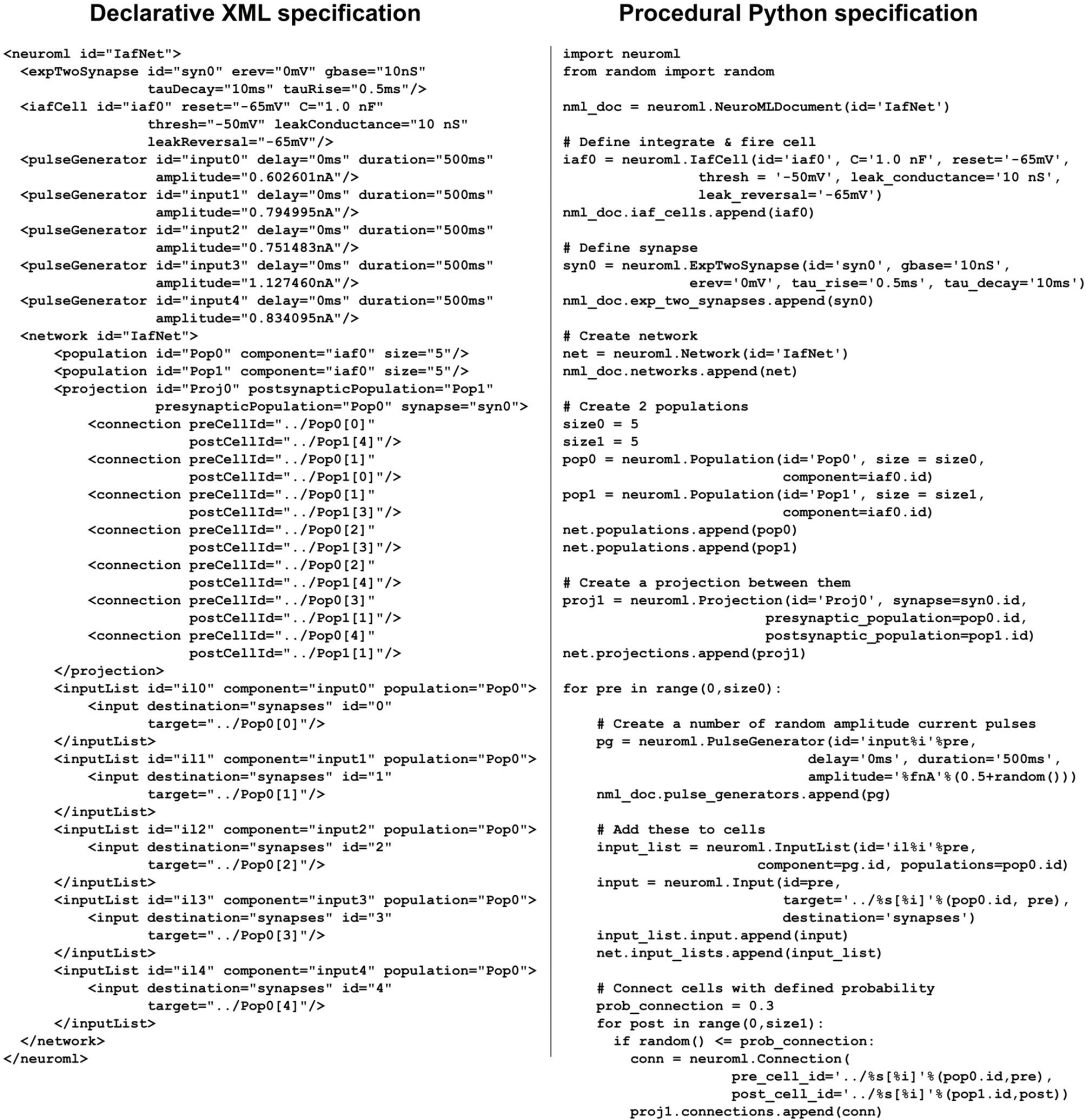
**Example of creation of a network of integrate-and-fire neurons using libNeuroML (right) and the equivalent XML representation (left)**. The network consists of two populations of five cells each. The cell type is a simple integrate-and-fire cell using the NeuroML element *iafCell*. A prototype component is created for this setting all of the parameters, along with a prototype synapse based on *expTwoSynapse*. A projection is created between the two populations and for each cell in the presynaptic population, an input current is applied, and connections are made to the postsynaptic cells with probability 0.3. The XML on the left is an example of one network instance which can be generated from this script.

libNeuroML can simplify the process of describing a model of a spiking neural network in NeuroML. All of the cell, synapse and channel models in NeuroML are available for use in networks, and the structure of a network can be generated in a procedural way, using all the capabilities of Python to encode the connectivity. Figure 7 shows a network of two randomly connected populations of integrate and fire neurons, one of which receives current injections of random magnitude. The structure of the network can be saved in XML format (or JSON format as outlined previously), but an important point is that many of the diverse network connectivity options available can not be described efficiently in NeuroML, and a Python script using libNeuroML provides a compact, cross-platform encoding of the network. While the XML in the example in Figure 7 is roughly the same length as the associated Python script, generally this will not be the case for larger networks. Also, the XML represents only one instance of a network that can be generated by the script. Modifying the script to specify the random seed will ensure reproducibility of the network connectivity, and so the libNeuroML version of the network can be distributed instead of the potentially very large XML or JSON serialization.

PyNN (Davison et al., 2009) is an API in Python that also offers the advantage of compact, procedural network descriptions. Currently, the cell models that can be defined in PyNN scripts are limited to a set of commonly-used point neuron models on simulators with PyNN backends. As outlined in the Discussion section of this article, we are actively working toward greater compatibility between PyNN and network descriptions in libNeuroML.

#### Multicompartmental cells

Aside from the ability to load and parse NeuroML documents containing morphological reconstructions of neurons, libNeuroML allows for the modification and analysis of these morphologies, as well as the generation of completely synthetic morphologies. Listing 1 shows an example of a script in libNeuroML which loads a morphology file from an XML representation, extracts the morphology and calculates the total volume and area of the segments in the cell. In this listing, **volume** and **area** methods do not have trailing parentheses because they are Python getter methods modified by the Python **@property** decorator. This demonstrates how helper methods can be used for operations that will commonly be required on those classes (e.g., getting the surface area or volume of the conical frustum). For details on how such helper methods can be added to libNeuroML the reader is referred to the libNeuroML developer documentation (http://libneuromldev.readthedocs.org/en/latest/#developer-documentation).

**List 1:**
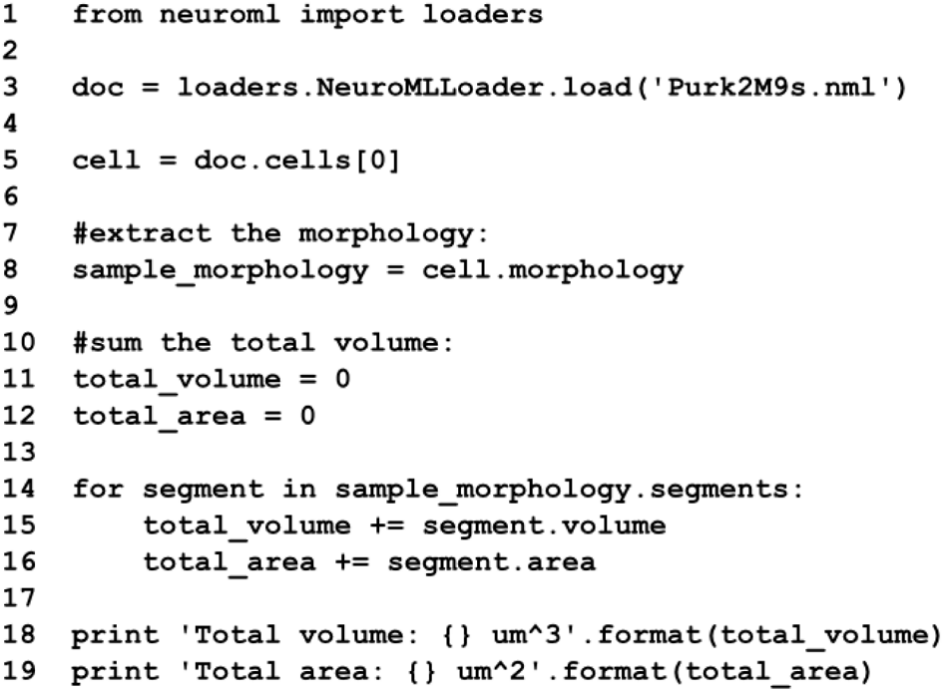
**Example of loading a NeuroML multicompartmental cell morphology with libNeuroML and using helper functions to calculate some of the properties (area and volume) of the cell**. Methods to calculate area and volume have been inserted into the code generated from the NeuroML Schema.

#### Openworm and neuroconstruct

The OpenWorm project (http://www.openworm.org) is an international collaboration with the aim of creating a physically- and biologically-detailed computer model of a behaving multicellular organism, the nematode *Caenorhabditis elegans*. The nervous system of this worm contains only 302 neurons, making it a very interesting model organism in experimental and computational neuroscience. The morphology of each of these neurons (as well as all other cells of the adult hermaphrodite) has been reconstructed in 3D at the VirtualWorm project (http://caltech.wormbase.org/virtualworm) and released into the public domain in Blender (http://www.blender.org) format, which is used for the creation of 3D applications. These have been converted to NeuroML format for the OpenWorm project and made available at https://github.com/openworm/CElegansNeuroML. Data on the connectivity between individual cells is available in a spreadsheet (CElegansNeuronTables.xls) in that repository and includes the numbers of known chemical or electrical connections between neurons of a particular type.

**Figure 8:**
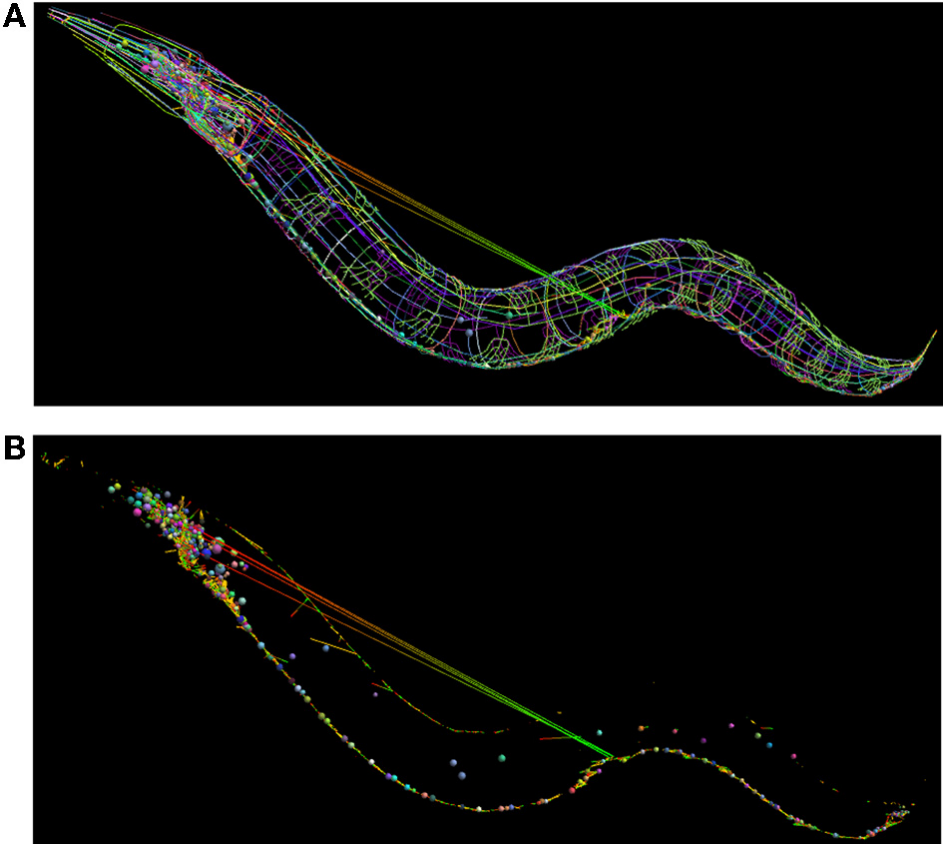
**Use of NeuroML and libNeuroML in the OpenWorm project. (A)** The full set of 302 neurons in the OpenWorm model are available in NeuroML format and are visualized here. Each cell, together with its spherical soma, is a different uniform color. Connections between neurons are shown as straight lines varying in color from presynaptic point (green) to postsynaptic point (red). These connections were generated by a Python script using libNeuroML based on connectivity data for pairs of cells. The long straight connections from cells in the midpoint of the worm to the head indicate an error in the connections in the data. **(B)** Same network as **(A)**, but only the somas of the cells, along with the synaptic connections are shown. The majority of connections are a short length due to the overlap between the neurites of the presynaptic and postsynaptic cells.

We have developed a libNeuroML-based Python script to analyze the integrity of this data that loads each neuron morphology, generates connections based the connectivity data, and saves the network file to NeuroML. When generating connections for each pair of cells where there exist N connections, the script chooses a variable number (100–5000 depending on number of segments in the cells) of random points on the presynaptic and postsynaptic cells and chooses the N closest pairs of points for the connections. The generated network file can be loaded into neuroConstruct (Gleeson et al., 2007), which can import NeuroML cells and networks for visualization (Figure 8). Due to the fact that all neurites are reconstructed, it should be possible to find points separated by a short distance on any connected pair of presynaptic and postsynaptic cells. The long connections between some neurons in Figure 8B (which could not be removed by increasing the number of random connections tested) highlights that these are unlikely to be real anatomical connections.

### PyLEMS example

**Figure 9:**
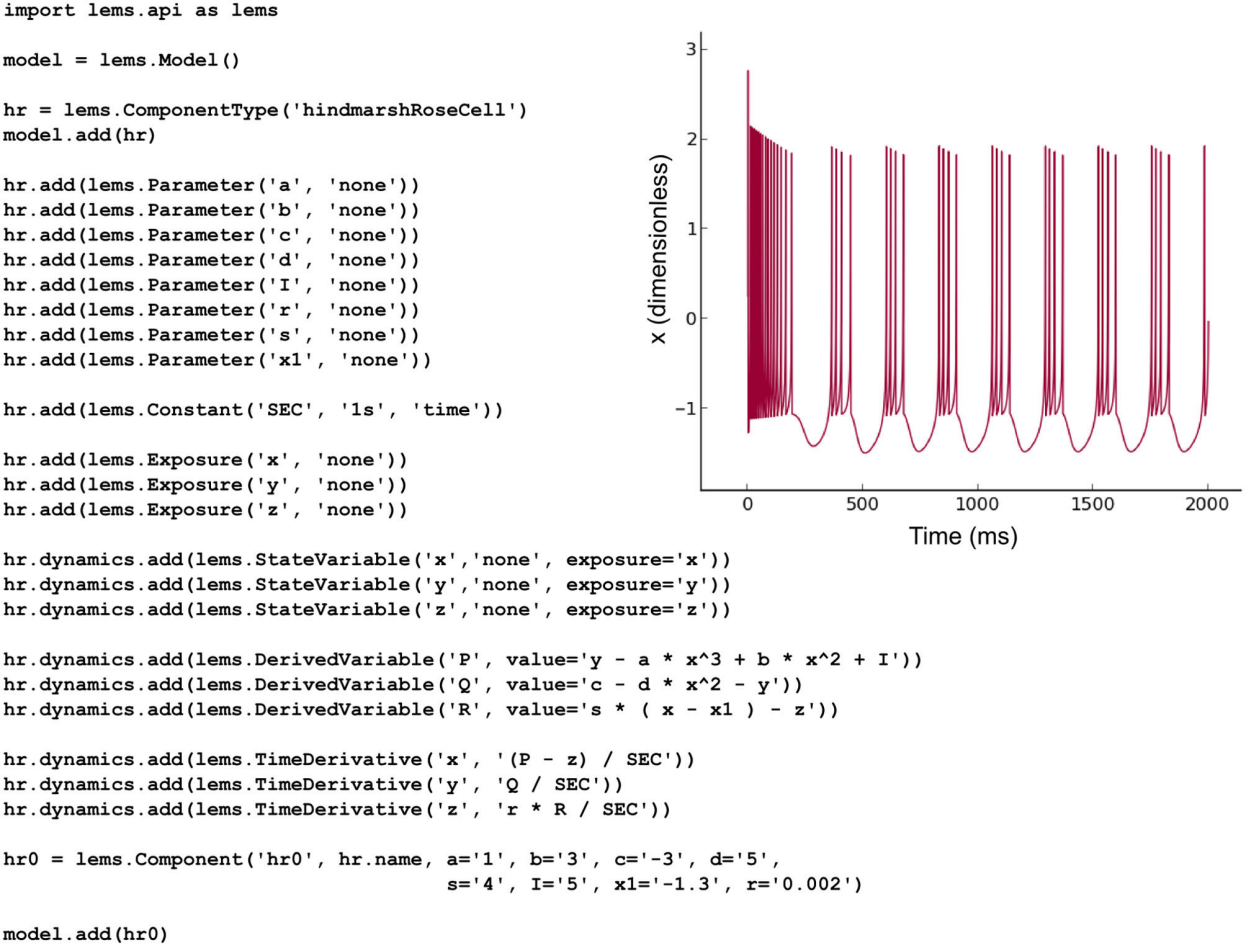
**The Hindmarsh and Rose spiking neuron model**. A LEMS *ComponentType* for this model is built adding the three state variables (x, y, z) and the eight fixed parameters required for instances of the model. Time derivatives for the state variables are specified using derived variables to shorten the required expressions. One *Component* with a particular set of parameters is created. This Python script produces valid LEMS XML which can be used by any LEMS-compliant simulator to simulate the model. The inset shows the behavior of the state variable x (corresponding to the model neuron's membrane potential) when the model is executed in PyLEMS.

The NeuroML v2 *ComponentType* definitions specified in LEMS for multiple commonly-used cell, synapse and ion channel types (Figure 2) can be loaded by PyLEMS. Another important feature of using LEMS as the basis for NeuroML v2 model types is that a modeler can define a new model type in LEMS if that model is not already present in the core definitions, significantly increasing the extensibility of the NeuroML language. PyLEMS allows these new models to be created using Python and saved in a valid, standard format. Figure 9 shows the code required to define a Hindmarsh and Rose spiking cell model (Hindmarsh and Rose, 1984) and the behavior of one state variable when simulated with PyLEMS. More details on the implementation of this model can be found on the Open Source Brain website (http://www.opensourcebrain.org/projects/hindmarshrose1984).

## Discussion

### Summary of advantages

NeuroML is a model description language which can represent biological concepts such as cells, ion channels and networks in a declarative format, while the corresponding mathematical and structural definitions of these elements can be defined in LEMS. The libraries described here, libNeuroML and PyLEMS, together provide a flexible toolkit for utilizing and extending the NeuroML language with Python. They complement the declarative model specification benefits of the XML forms of these languages by offering APIs which can be used for procedural model development. While the libNeuroML API provides a Python object model which has a type interface defined by the NeuroML Schema, it also offers a memory-efficient array-based internal representation for morphological neuron reconstructions and allows export of NeuroML models into different formats. libNeuroML also provides a range of helper methods that provide several common operations required when interacting with models expressed in NeuroML. In the same way that libNeuroML provides access via Python to NeuroML, access to the LEMS language is provided by PyLEMS. While Python interfaces exist for many neuronal simulators, giving access to their native object model (e.g., NEURON Carnevale and Hines, 2006) or allowing new neuron models to be specified (e.g., Brian Goodman and Brette, 2008), this is the first effort at a comprehensive Python suite allowing standards based specification of neuronal model elements from ion channels and synapses to complex networks of cells in 3D, accompanied by programmatic access to underlying model definitions.

Another advantage of these Python APIs is that they provide a way to add functionality that is not provided in the declarative representation. In particular, the current version of NeuroML focuses on the expression of individual, instantiated networks and is less useful for expressing the many probabilistic rules which could be used for network creation. This can be overcome by implementing the network creation process in Python and instantiating the resulting model through the API. Greater support for declarative network templates, including network connectivity rules, is under development for NeuroML 2, but the language is unlikely to match the full flexibility available through a programmatic interface.

### Interaction with PyNN and other initiatives

A complementary initiative to create a Python API for simulator-independent model specification is PyNN. This API has traditionally concentrated on allowing scripts for large-scale network models with point neurons to be written once and used across multiple simulators with Python interfaces which support these types of model, such as NEST (Gewaltig and Diesmann, 2007), NEURON (Hines and Carnevale, 1997; Carnevale and Hines, 2006; Hines et al., 2009) and Brian (Goodman and Brette, 2008). While the focus of NeuroML v1.x was on multicompartmental, conductance-based cell models, the wider scope of NeuroML v2 has led to increased overlap with PyNN. There are LEMS definitions for the dynamical behavior of the standard PyNN neuron models and corresponding NeuroML elements for these (e.g., *IF_cond_alpha* and *EIF_cond_exp_isfa_ista* in Figure 4), and there is an experimental implementation which allows PyNN scripts to export a full model description in NeuroML v2 format. For example, whereas **from pynn import nest** at the start of a PyNN script indicates that the model should be executed in NEST, **from pynn import neuroml2** indicates that the script should save a declarative description of the model in NeuroML. In addition, code generation to allow all cell/synapse types in NeuroML to be used in PyNN scripts is under active investigation.

NineML (Raikov et al., 2011) is a language for describing new models of spiking neurons in a machine-readable format, which has been developed in parallel to LEMS. NineML shares a number of features with LEMS, allowing new models of spiking neurons to be specified (in the Abstraction Layer). However, a key advantage of LEMS is the close interaction with NeuroML 2, allowing modellers to choose to use either of the languages independently, or to make use of the curated sets of LEMS definitions of standard model types available for NeuroML 2. Creating hierarchical models is also difficult in NineML, but is a key feature of LEMS, required for specifying complex conductance based cell models and ion channels. SpineML (Richmond et al., 2013) is a language that has recently been derived from NineML and shares many of its design choices. LEMS to NineML and SpineML conversion is a feature of the Java based jNeuroML tool (see below), which will assist interoperability between these languages.

### libNeuroML as an object-model for third-party applications

One potential use of libNeuroML is to provide an object model for third-party applications such as visualization, modeling, and simulation libraries. This is the route taken by the in-development Pyramidal project (http://pyramidal.readthedocs.org), which seeks to create an API for running multicompartmental simulations across different simulators. In contrast to libNeuroML, which loads the model elements into an internal object tree for manipulation, Pyramidal interacts with the underlying simulator to store the model in that application's native format. The advantages are that the modeler only deals with model elements defined in NeuroML and interacts with them through libNeuroML, while the simulator uses its own efficient, internal representation, and scripts developed in this way are portable across simulators.

This approach of using libNeuroML as a library could be used for other applications which only use a subset of the elements of NeuroML, such as an application for visualizing or editing neuronal morphologies or an application for analyzing channel kinetics.

### Java libraries for NeuroML and LEMS

In addition to the Python APIs described here for working with NeuroML and LEMS, there are corresponding libraries in the Java language for reading, editing, writing, and validating NeuroML (https://github.com/NeuroML/org.neuroml.model) and LEMS (https://github.com/LEMS/jLEMS) documents. jLEMS is a more mature package than PyLEMS and is the reference simulator implementation of the LEMS language. These libraries are complemented by other Java packages for importing and exporting multiple formats such as SBML, NEURON, and Brian into LEMS using code generation. A tool jNeuroML (https://github.com/NeuroML/jNeuroML) exists which bundles the functionality of these Java packages, allowing easy access to these features from command line. Options for allowing some of these code generation features to be accessible from Python are under investigation.

### Future work

Benchmarks indicate that HDF5 is a potentially powerful tool for improving the performance of libNeuroML read/write operations; however, currently, HDF5 can be used to serialize only a limited subset of components defined in NeuroML (reconstructed morphologies). A future avenue of research is the extension of libNeuroML HDF5 serialization to support full NeuroML documents. Additionally, none of the helper functions provided by libNeuroML assist the user with the creation of models of neural networks; we are planning to include this functionality in future versions of libNeuroML.

It should be noted that there are some limitations to the ability of LEMS to describe all models within the scope of NeuroML v2. Currently LEMS does not permit a fully machine-readable, simulator independent description of the equations to solve for neurons with more than one segment, i.e., compartmental modeling. This is planned for future extensions of LEMS.

## Conclusion

NeuroML and LEMS are key languages which enable cross-simulator portability of models and increase the accessibility and transparency of model properties. Python libraries for reading, writing and manipulating models in these languages are an important step toward encouraging the wider use of these languages. Making stable versions of these libraries available to the computational neuroscience community is and will remain a core part of the release process for NeuroML.

### Conflict of interest statement

The authors declare that the research was conducted in the absence of any commercial or financial relationships that could be construed as a potential conflict of interest.
